# Correspondence

**Published:** 1875-06

**Authors:** C. M. Wright

**Affiliations:** Basel, Switz.


### Correspondence.

BASEL, SWITZ., Feb. 11, 1875.

*Dear Doctor Smith*:

As it is nearly time for the old Mississippi Valley Association to meet, perhaps a word from an old member now in an old country would not be taken amiss. After about three years of trial, of experience of dentistry in a foreign land, perhaps my opinions might be interesting about a life of a dentist here and the practice (of American Dentistry) abroad. We receive letters often from young dentists asking about the prospects, older and well established dentists too, often apply. They get a little discontented at home, get tired of everlasting routine of dental practice at home, find that money don't flow in fast enough and being discontented they remember the flattering accounts they have heard of Dr. Tom Dick or Harry who went to Europe and made an immense fortune. Now down in their hearts they feel that Dr. Tom or Dr. Dick whom they know are not or were not quite as good dentists as they themselves are and yet to think of these Doctors going to Europe, representing American Dentistry and making a fortune. So the discontented fellow at home sighs for Europe or South America or China. These reports are very much like the accounts of great nuggets of gold being found in California and a man made rich in a day. They grow on a man, they haunt him at night, they float before his eyes as he labors at home, they come between him and his filling of gold. His mind becomes displeased and if he has great courage he gives up his home and hies for Europe. Ho! when he arrives if he has burnt his ships behind him, he stays with a great disappointment, a great sense of having been sold, hovering over him, and one by one the dreams, the prospscts, once so enticing mock at him and he cries "it is all a delusion and a snare" 1st He is cut off from the agreeable social life of America and remains a pilgrim and a stranger, a foreigner with no place, no part in the social life abroad. He is no longer the "rich American Traveler" but a dentist. 2d. He loses all the professional stimulus so abundant in America his dental societies his dental friends. 3d. He is no longer practically a citizen and he feels the loss of the privileges he did not value at home. 4th. He finds dental work, a dental office about the same as at home. Teeth are just as bad no matter what the the theory at home is, as in America; and he must fight against prejudices, and while he is educating his patients up to his standard, must also modify his practice to meet a different atmosphere, a different mind among his patients, 5th, Now for the fortune. Ah, you say, what if a man loses all these foregoing things and gains in a few years fortune's sweet smile? Yes, but suppose he sells his soul and then don't get the fortune. What a miserable chap he will be. Fortunes in dentistry can be acquired here just as they can at home. If a man has a comfortable practice, collects his fees and spends nothing he may be rich after a while at home or abroad, but to live moderately and like a professional gentleman at home or abroad costs money and I have not seen an instance of the cheapness of Europe, that has not its contrapart at home. That's so. Let no man who spends from $3000 to $4000 dollars at home for liveing, expect to spend less in Europe. Let no man who by economy spends but $1200 or $1500 at home expect to do the same for one franc less in Europe. If you will live on cheese and bread and beer at home you can be rich, but if you educate your children, clothe yourself and family well, live like a gentleman, as most dentists do or try to, don't expect to do it on one cent less in Europe than in America. I have not seen the wealthy dentists of Europe, and I have met a good many dentists. I have, however, seen several good fellows laboring away for a living and privately swearing at themselves for ever having been such fools as to leave dear old America.

Let me tell how to get a practice in Furope, one way is to go and open an office and sit down in it as at home and wait for business. It will come with patience and intelligence applied in the waiting, as surely as it will in America. The other way is to buy at the fanciest kind of figures "a dental practice" and this is the best way, for after you have made payments on your practice you must stick, you can't afford to be discouraged and no matter if you find you have bought what the Ohio boys vulgarly call "pig in a poke," you've paid some on the pig and you hope the pig will come to you. So you stick and sticking is one of the elements of success.

Before a practice is bought it is magnifique, glorious; after it is yours, its a struggle and a fight for life as at home until you have made it yours, by the ordinary and usual ways of making a reputation and proving yourself a success, which could be as easily done at home. These are unfortunately facts. The theories have been laid on the shelf. Are there no bright sides to European practice. Oh! certainly. The people are generally polite and agreeable as patients, and follow your advice better than American patients. They pay their bills, but can not appreciate the high prices which certain works of art must command. They do not boast as some do at home. "Ah, I regard my dentist as a genius. I've got $500, $700 or $1000 worth of his work in my mouth," etc., etc. 2d. We have excellent servants and housekeeping is easier than in America. 3d. We have the ever present hope of going home, and this last I think may be said to be the brightest side of European practice. Come over, brothers, and try for yourselves. God bless the Mississippi Dental Association and you and my dear old friends.

Yours as ever, C. M. Wright.

